# Transportation Network with Fluctuating Input/Output Designed by the Bio-Inspired *Physarum* Algorithm

**DOI:** 10.1371/journal.pone.0089231

**Published:** 2014-02-26

**Authors:** Shin Watanabe, Atsuko Takamatsu

**Affiliations:** Department of Electrical Engineering and Bioscience, Waseda University, Shinjuku-ku, Tokyo, Japan; National Research & Technology Council, Argentina

## Abstract

In this paper, we propose designing transportation network topology and traffic distribution under fluctuating conditions using a bio-inspired algorithm. The algorithm is inspired by the adaptive behavior observed in an amoeba-like organism, plasmodial slime mold, more formally known as plasmodium of *Physarum plycephalum*. This organism forms a transportation network to distribute its protoplasm, the fluidic contents of its cell, throughout its large cell body. In this process, the diameter of the transportation tubes adapts to the flux of the protoplasm. The *Physarum* algorithm, which mimics this adaptive behavior, has been widely applied to complex problems, such as maze solving and designing the topology of railroad grids, under static conditions. However, in most situations, environmental conditions fluctuate; for example, in power grids, the consumption of electric power shows daily, weekly, and annual periodicity depending on the lifestyles or the business needs of the individual consumers. This paper studies the design of network topology and traffic distribution with oscillatory input and output traffic flows. The network topology proposed by the *Physarum* algorithm is controlled by a parameter of the adaptation process of the tubes. We observe various rich topologies such as complete mesh, partial mesh, Y-shaped, and V-shaped networks depending on this adaptation parameter and evaluate them on the basis of three performance functions: loss, cost, and vulnerability. Our results indicate that consideration of the oscillatory conditions and the phase-lags in the multiple outputs of the network is important: The building and/or maintenance cost of the network can be reduced by introducing the oscillating condition, and when the phase-lag among the outputs is large, the transportation loss can also be reduced. We use stability analysis to reveal how the system exhibits various topologies depending on the parameter.

## Introduction

Transportation networks such as power grids are, in general, designed under certain static supply-demand conditions. However, in most situations, whether the network is that of nature or a man-made system, the inputs/outputs into/from the networks fluctuate rather than remain constantly static. One such example is an ant foraging trail network, in which ants cannot constantly prey upon their foods because the activities of the prey animals fluctuate daily or seasonally. The feature also holds for man-made networks. For instance, the number of passengers commuting by rail is maximized in the mornings and evenings and the peak times shift among stations in suburbs and city areas on weekdays. Additionally, the transportation patterns on weekends are quite different from those on weekdays. The second man-made example is power grids. The pattern of electricity consumption is distributed according to the lifestyles or business style of consumers, which was recently confirmed using clustering analysis on a town in Japan [1]. More specifically, the consumption pattern fluctuates daily, weekly, and seasonally, and the peak time depends on the consumers.

Optimization of networks under fluctuating conditions is difficult to be conducted in a straightforward manner by conventional methods within linear- and nonlinear-programming frameworks. In this paper, we propose designing traffic distribution in networks under fluctuating conditions using an algorithm inspired by the organism *Physarum*.

The *Physarum* algorithm, which mimics the shortest path-finding behavior of the plasmodial slime mold organism [2], formally called *Physarum polycephalum*, was developed by Tero et al. [3]. The plasmodium of *Physarum* is a giant amoeba-like multinucleated unicellular organism. It contains thousands of nuclei, so the cell size can get very large, ranging from 10 µm to 1 m. To distribute protoplasm, including nutrients, oxygen, and organelles, throughout this large cell body, the organism has developed a peculiar transportation network consisting of tubular structures. The diameter of the tubes adapts to the flux of the protoplasm: The tubes on the paths connecting multiple food sites become thick in accordance with the growth of the protoplasmic flow, while the other paths become thin and finally disappear when there is little or no flow. Consequently, the organism is able to generate the shortest paths connecting multiple food sites [2], [4]. The *Physarum* algorithm, which mimics the adaptive behavior of the tubes, has been widely applied to complex problems such as maze solving [2], design of the topology and transportation distribution of railroad grids [5], [6] and highway networks [7], and path formation in wireless sensor networks [8]. Although, in the above examples, it was applied under static conditions, the algorithm can also be applied under fluctuating conditions owing to its adaptive behavior.

This paper studies the design of network topology and traffic distribution under oscillating conditions, which is the simplest type of fluctuating environment. The network consists of nodes and links, which, in power grids for example, correspond to consumers, power plants, electric poles, and power lines. A multiplicity of consumers uses electricity with daily periodicity (oscillating condition). The peak consumption times vary according to the consumers, and are defined by phase lags.

In the Methods section, we outline how the *Physarum* algorithm is modified to deal with problems involving oscillating conditions and define performance functions. We then present the network designs under oscillating conditions proposed using the *Physarum* algorithm and evaluate them using our performance functions, in the Results section. In the Discussion section, a stability analysis for a simple network is considered in our discussion of the numeric result. Finally, we discuss the effect of the oscillating condition and the phase lags.

## Methods

### *Physarum* Algorithm

**Figure 1:**
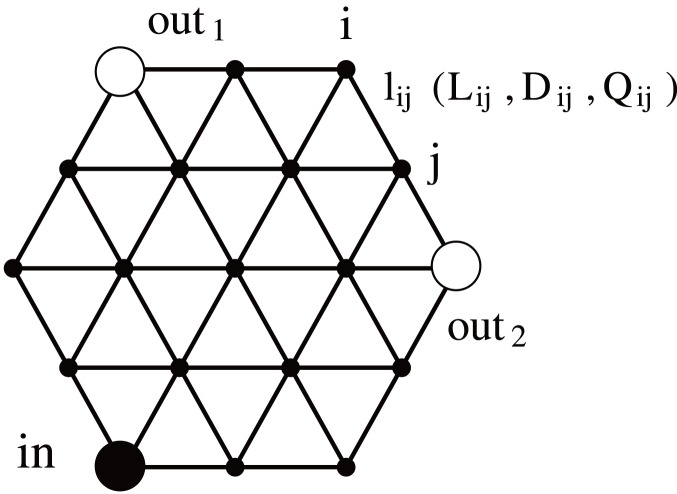
Network topology used by the *Physarum* algorithm for numerical calculation.

In this section, we modify the original *Physarum* algorithm [3] to deal with the example network depicted in Fig. 1. The shaded and unshaded large circles, respectively, represent nodes for input (denoted as *in*) and output (denoted as 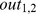) of transported materials, such as protoplasm in *Physarum*, current in power grids, and people in railroad grids. The link 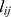 connecting the nodes 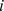 and 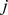 has the following properties: length 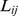, conductivity 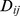, and traffic volume flux 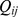. Their meanings in each application are summarized in Table 1.

**Table 1:** Correspondence of variables.

Variables	General	*Physarum*	Power Grid
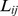	length of link	length of tube	length of electric wire
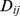	conductivity	tube thickness ( 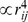 )	electric conductivity ( 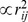 )
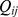	flux of traffic volume	flux of protoplasm	current

The radius of tubes and wires are represented with 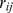.

The flux at each node conserves
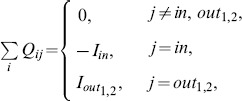
(1)where 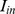 and 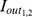 are the fluxes at 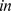 and 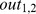, respectively. The total flux to/from the system should be balanced:



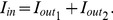
(2)The flux 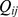 is given by
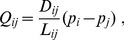
(3)where 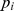 and 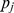 represent, respectively, the pressure at nodes 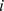 and 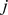. Substituting Eq. (3) for Eq. (1), 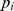 is obtained under the given 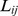 and 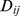. 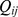 is then calculated using Eq. (3) again. In the numerical calculations, 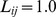 is set at all links.

As mentioned in the introductory section, the conductance 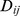 adapts to flux. Therefore, the conductance is assumed to evolve according to the following differential equation:
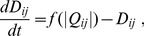
(4)meaning that the tube grows depending on the flux (the first term on the right hand side of the equation) while it degenerates (the second term). It is natural in biological systems for the growth rate to be saturated by an upper limit. Thus, function 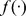 can be defined as a sigmoid function:



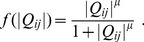
(5)This function is widely found in biological cooperative processes [9]. The parameter 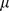 is the key parameter governing the dynamics of this system. When 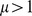, the tube grows only slightly when the flow is extremely weak, although the growth speed is accelerated when it once starts to grow, then it is finally saturated at one. When 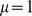, the function is categorized into the Michaelis–Menten type, which represents the simplest enzymatic reaction. When 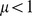, 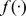 represents fast initial growth and slow saturation, suggesting no meaning related to biological processes.

### Application to Networks with Oscillating Conditions

The outputs at 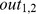 are assumed to oscillate as follows:
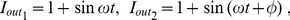
(6)where 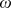 is angular frequency and 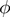 represents a phase lag between the outputs. In Eq. (6), 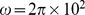 is assumed so that the period of oscillation 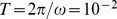 is small enough to the time constant of the degeneration process of 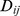, which is estimated as one. We confirmed that the outline of the results in this paper is valid over the frequency range 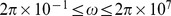, namely, over the period range 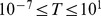 (see 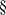
1 in File S1 for details).

### Converged Value of 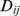


We repeat the computation of Eq. (4) with Eqs. (1)–(3), and Eq. (6) until 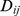 converges within a certain accuracy. In fact, 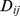 continues to oscillate slightly even after a long evolution period (Figs. A and B in 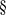
1 in File S1). Therefore, the completion of the convergence is judged according to the following criterion.

**Figure 2:**
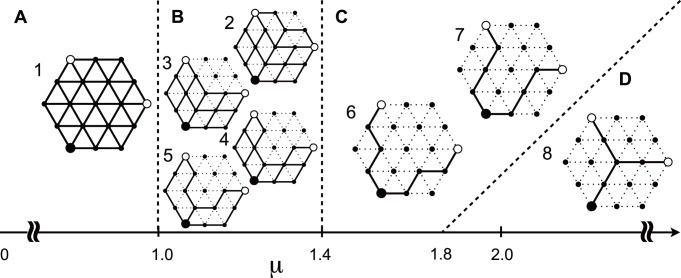
Dependence of network topology under constant input and output. Initial values of 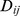 were either set as homogeneous (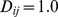 for all links) or were distributed according to a normal distribution with mean 1.0 and standard deviation 0.1. Solid and dashed lines of the network diagrams denote surviving and removed links, respectively. **A** Complete mesh (type 1), 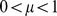. **B** Partial mesh (types 2–5), 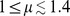. **C** V-shaped network (types 6 and 7), 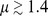. **D** Y-shaped network (type 8), 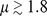. When the initial conditions of 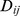 are exactly homogeneous, the V-shaped network appears in the range of 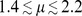 and the Y-shaped network appears in the range of 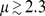. Type numbers correspond to those of Figs. 3 and S1.

**Figure 3:**
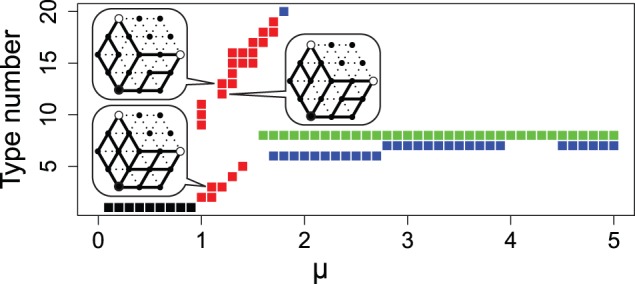
Network types calculated with oscillating inputs and outputs. A type number is assigned to each topology. The full list of network topologies is represented in Fig. S1. The data are those for the homogeneous initial conditions of 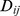. The plots of mesh, partial mesh, V-shaped and Y-shaped networks, are colored in black, red, blue, and green, respectively. The dependence of type of partial mesh on 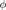 is shown in Fig. S2.

First, a variation amount of 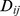 at time 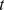 is defined as follows:
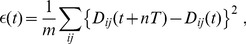
(7)where 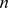
(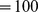) is the number of cycles in the input/output oscillation, and 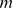 is the total number of links. The convergence of 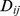 is judged to be complete when 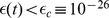 (the value of 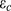 is set by the reasoning below). Consequently, the averaged value 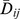 at time 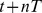 is calculated using the following definition:



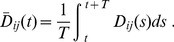
(8)After the convergence is ascertained, 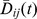 is denoted as 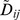. The link is removed (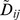 set to zero) when 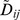 becomes less than a certain threshold 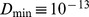. The value is sufficiently smaller than the order of the maximum value of 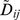 (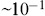). Finally, the network topologies and traffic distributions (magnitude of conductances) recommended by the *Physarum* algorithm are obtained (Figs. 2 and 3). Note that the threshold value for judgment of 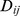-convergence 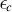 is sufficiently smaller than 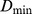.

### Performance Functions

We now introduce three performance functions to evaluate the performance of the networks recommended by the *Physarum* algorithm: power or transportation loss, building and/or maintenance cost, and vulnerability in network topology.

Loss 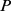 is defined using an analogy to electric energy loss, which is calculated with 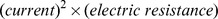 in a wire. Consequently, the loss for a link 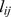 is defined as 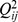 multiplied by 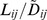 (see also 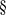
2 in File S1). The total loss for the network is calculated by summing the loss for each link over all the links as follows:
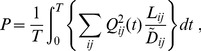
(9)where the loss is averaged over a period of input/output oscillation because 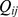 oscillates.

Cost 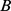 is that for building and/or maintaining a network, which is expected to be proportional to the total volume of the network. Because the cross section of each link is proportional to 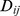 in the case of power grids, as described in Table 1, 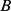 is defined as follows:
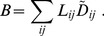
(10)


Note that 
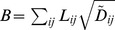
 should be adopted when considering the original *Physarum* network because the relation between conductivity and a tube of radius 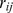 is described as 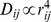 (see also Table 1) [3], [6].

Vulnerability 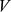 is defined as the probability that the connection 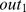 or 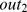 from 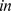 is divided when one of the links in the network is randomly deleted. The deletion frequency is assumed to be proportional to the length of the link when 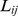 is not homogeneous, where the probability is normalized by the total length of the network, 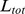. Consequently, the vulnerability is defined as follows:
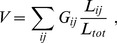
(11)where disconnectivity 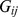 for a link 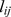 is set to one if transportation flows out from 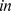 can reach neither 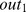 nor 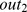; otherwise, it is set to zero.

## Results

We considered two cases, constant and oscillating flux at input and output nodes, and evaluated the network topologies and traffic distributions recommended by the *Physarum* algorithm using the three performance functions.

### Constant Condition

Before capturing the effect of oscillatory input/output on the network design, we tested the effect with constant input/output. We set the fluxes to constant values, 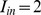, 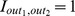, in Eq. (1). The numerical calculation started from a homogeneous initial condition of 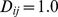 or a non-homogeneous condition according to normal distribution with mean 1.0 and standard deviation 0.1. We observed eight types of network topologies in the parameter range 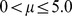 as shown in Fig. 2.

The network topology changes from dense to sparse depending on 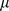. When 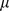 is smaller (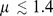), the network forms a mesh accompanied by circular structures (Figs. 2**A** and 2**B**). When 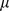 becomes larger (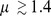), the network forms a tree structure (Figs. 2**C** and 2**D**). The mesh networks are categorized into two types, complete mesh (type 1; Fig. 2**A**) and partial mesh (types 2–5; Fig. 2**B**). The tree networks are categorized into two types, V-shaped (types 6 and 7; Fig. 2**C**) and Y-shaped (type 8; Fig. 2**D**) networks. The Y-shaped networks appear when 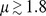. The paths from the input are partially shared in the Y-shaped network, while they are directly connected to the two outputs in the V-shaped network.

### Oscillating Condition

We set the input/output flux oscillating using the definition in Eq. (6). The numerical calculation started from a homogeneous initial condition of 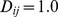 or non-homogeneous conditions according to normal distribution with mean 1.0 and standard deviation 0.1. We observed 20 types of network topologies in the parameter range 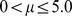, as shown in Figs. 3, S1 and S2. In this case, the dependence of network topology on 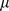 is similar to that of the constant condition: when 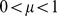, complete mesh (type 1) appeared. As 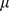 increased over 1, the topology changes to partial mesh (types 2–5 and 9–19). Finally, when 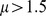, V-shaped (types 6 and 7) or Y-shaped (type 8) networks were observed. It should be noted that the variation of the topologies becomes broader than in the case of the constant condition when 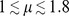: a variety of partial meshes, i.e., networks of types 9–19 besides types 2–5, were observed.

**Figure 4:**
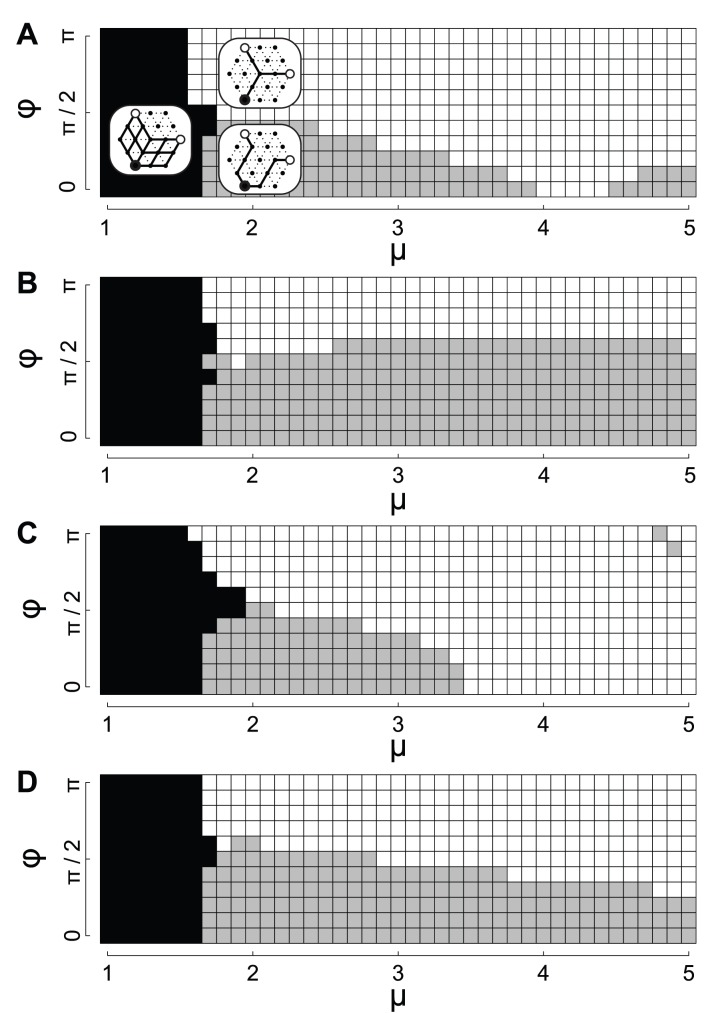
Relation between network types and the parameters 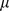 and 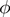 under oscillating conditions. 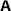
 Homogeneous initial condition of 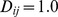 . 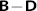 Examples of non-homogeneous initial condition of 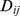: Initial values of 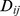 were distributed according to a normal distribution with mean 1.0 and standard deviation 0.1. Black, gray, and white squares denote partial mesh, V-shaped and Y-shaped networks, respectively. The specific type-number of partial mesh depends on both parameters 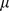 and 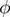 (Fig. S2), and also on the initial condition of 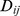, which is not shown here in detail.

The network topology depends not only on 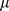 but also on phase lag 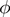 and on the initial conditions of 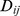. The characteristics are particularly evident in 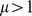. Figure 4 shows the network types observed according to 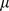, 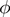, and the initial conditions of 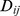. For 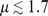, primarily partial meshes were observed (see Fig. S2 for details). Dependence of the topology on 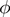 can be seen more clearly when 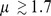: the V-shaped network is more frequently observed when the two outputs are in phase (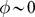) and the observation ratio of the Y-shaped network increases accordingly as the lag approaches anti-phase (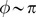). Note that the dependence of the topology on 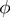 is also subject to the initial distribution of 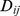 in detail (compare the diagrams **A** of homogeneous condition and **B**–**D** of three different non-homogeneous conditions in Fig. 4).

### Evaluation of the Networks

**Figure 5:**
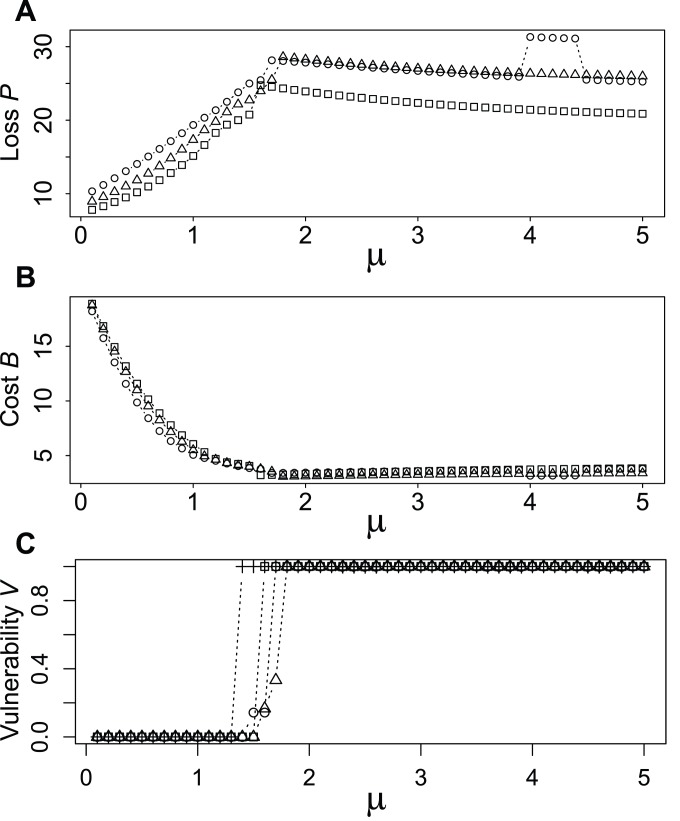
Performance depending on parameters 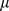 and 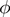. 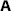
 Loss 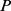. 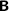 Cost 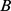. 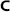 Vulnerability 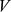. Circles, triangles and squares denote performances when 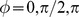, respectively. The crosses in **C** denote the performances of the constant condition. The data are those for the homogeneous initial conditions of 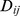. The case starting from non-homogeneous initial conditions is demonstrated in 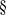
3 in File S1.

Figure 5 shows the performances 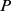, 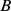, and 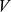 estimated for each combination of parameters 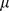 and 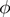, where each network is calculated from the homogeneous initial conditions of 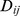. Smaller values mean better performances in these analyses. Loss 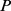 increases until around 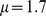, then slightly decreases, irrespective of 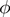, as shown in Fig. 5**A**. Notably, 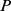 for 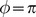 is clearly always smaller than those for 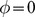 and 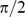. The discontinuity in the plots for 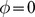 when 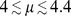 is caused by the discontinuous change of network topology. Cost 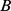 decreases rapidly until around 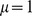, then it becomes almost constant, as shown in Fig. 5**B**. Vulnerability 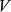 equals 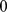 when 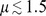, as shown in Fig. 5**C** because the network includes circular structures (Figs. 2**A and B**). As 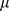 exceeds around 1.5, 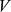 jumps to 1.0 because the network includes no circular structure. In conclusion, the network is well balanced at 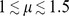. The results for the non-homogeneous initial conditions of 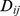 are valid for virtually the same feature as in the case for the homogeneous conditions (see 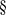
3 in File S1 for details).

### Benefit Derived from the Introduction of Oscillatory Condition

**Figure 6:**
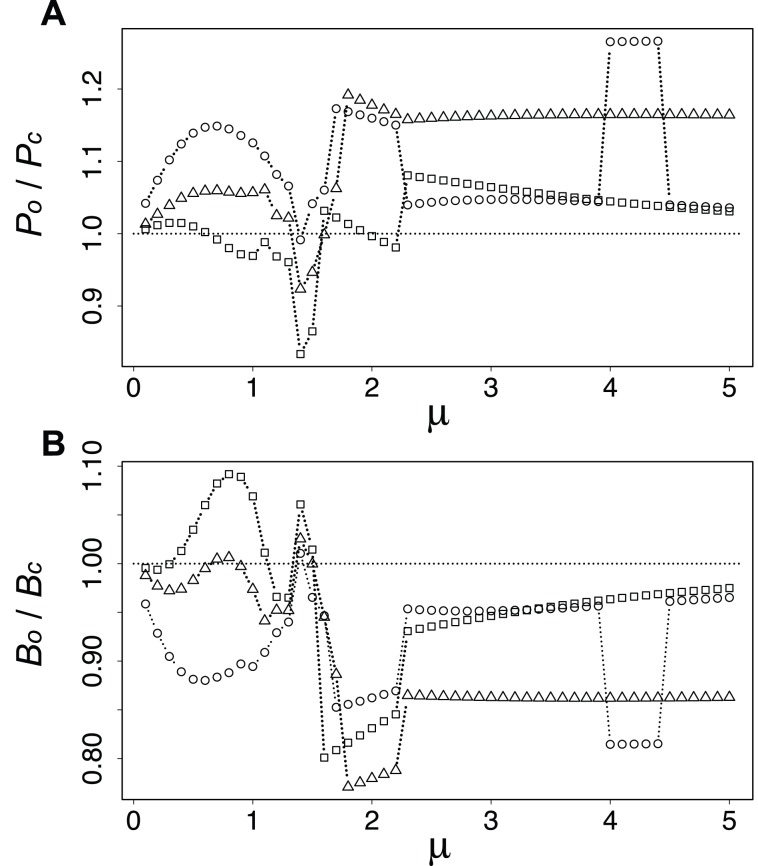
Comparison of the performances for the networks designed under constant and oscillatory conditions. **A** Ratio in loss, 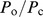. **B** Ratio in cost, 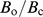. Circles, triangles, and squares respectively denote 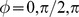. The data are those for the homogeneous initial conditions of 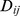.

To investigate the benefit derived from the introduction of the oscillatory condition, we calculated the ratio of the performances between the constant and oscillatory input/output, as shown in Fig. 6. Note that the performance 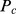 and 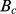 were estimated with oscillatory input/output against the networks obtained under constant condition by the *Physarum* algorithm. The performances 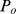 and 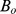 were estimated with oscillatory input/output against the networks obtained under the oscillatory condition, which are the same as those of Fig. 5.

A ratio with value smaller than 1.0 suggests that the performance of the network considering the oscillatory condition is better. Loss 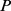 for the oscillatory condition is better than that for the constant condition only when 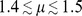. In contrast, cost 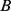 almost always shows better performance in the oscillatory condition. The cost can be reduced to about 80% in the best performance. The effect of vulnerability is captured in Fig. 5**C**: Vulnerability is improved by considering the oscillatory condition when 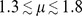.

## Discussion

### Stability Analysis of Network Topology

**Figure 7:**
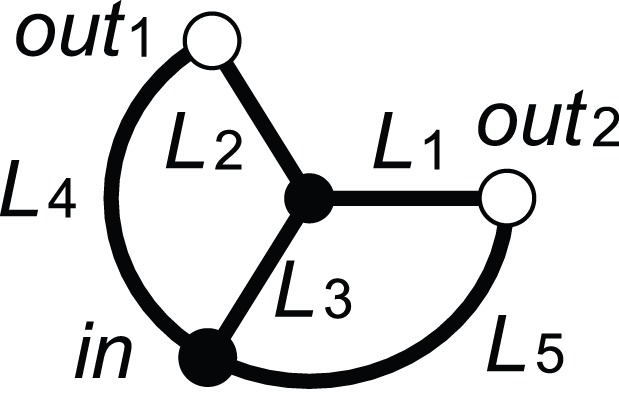
The simple network used for stability analysis. The link lengths were set as 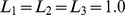 and 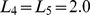 so that any path length from 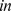 to 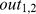 is 2.

To understand the parameter dependence of the network topology, we conducted stability analyses of network topologies and estimation of their basin size against a network with small compositions of nodes and links (Fig. 7).

In this subsection, the notation of link 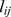 is redefined as 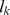. In accordance with this definition, the equations for conductances 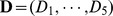 are rewritten instead of using Eq.(4) as follows:
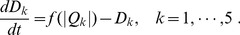
(12)


The equation (6) is redefined as 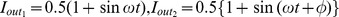, where the magnitude of the input/output flux is set as half of those in Eq. (6) because the network size is now reduced.

In Eq. (12), 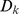 has two time scales: slow and fast. The fast time scale is caused by 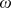, which gives fluctuations with small amplitude to 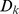. Accumulation of the small asymmetric fluctuations finally derives a slow drift in 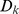. The final network topology must be determined mainly by the slow dynamics. Therefore, 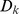 can be averaged over a period of the fast dynamics when we focus only on slow dynamics, which is denoted as 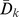 hereafter. The slow dynamics of 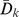 can be written as follows:
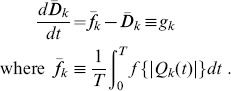
(13)


The steady state of Eq. (13), 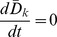, is considered then the solutions of the equation 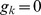, namely equilibria, are denoted as 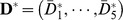. The magnitudes of individual elements of 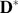 determine the topology of the network. Note that 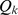, and also 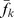, are a function of 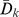 owing to Eq.(3). Therefore, we solved equation 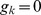 using Newton’s method, where 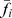 is obtained by numerical integration of 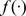 according to the above definition using Eqs. (1)–(3), (5). The integration of 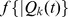 over the period of output oscillation in Eq. (13) depends on 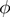 because of the nonlinearity of the function (see 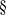
4 in File S1 for details).

**Figure 8:**
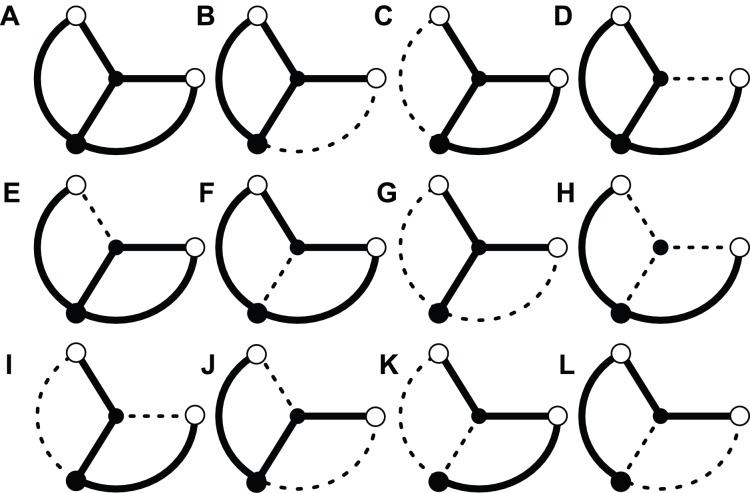
Twelve equilibria for the network in Fig. 7 represented in network-topology form. **A** Complete mesh, **B–F** partial mesh, **G** Y-shaped network, **H–J** V-shaped network, **K** and **L** the others.

**Figure 9:**
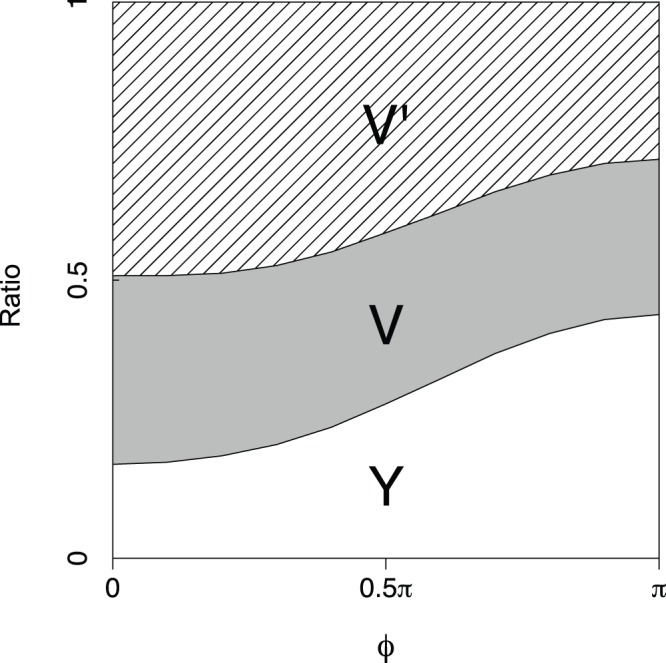
Observation ratio for small network. The network in Fig. 7 was used. Y, V, and V′ respectively denote the network topologies represented in Figs. 8**G**, 8**H**, and 8**I–J**. The parameter 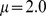 was set. In each calculation against 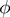, all combinations among 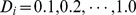 for all links 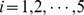 (specifically, a total of 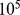 combinations) were tested as initial conditions. The networks such as the ones shown in Fig. 8**K** and **L** were also observed but the observation ratios were extremely small, e.g., 0 when 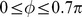, 0.002 when 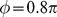, 0.018 when 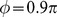, and 0.03 when 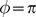.

We obtained 12 equilibria of 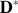, as summarized in Fig. 8, where the topologies are drawn based on the magnitude of the elements’ values, 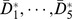. The topologies can be roughly classified into complete mesh (Fig. 8**A**), partial mesh (Figs. 8**B**–**F**), Y-shaped (Fig. 8**G**), V-shaped (Figs. 8**H**–**J**) networks, and others (Figs. 8**K and L**) similar to those of Fig. 2 and S1. The V-shaped network is, furthermore, divided into subcategories: symmetric (Fig. 8**H**, denoted by the V-shaped network in Fig. 9), and asymmetric (Figs. 8**I**–**J**, denoted by the V′-shaped network in Fig. 9).

We conducted linear stability analysis for each equilibrium 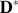. The Jacobian matrix 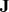 of 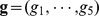 is defined using Eq. (13) as follows:
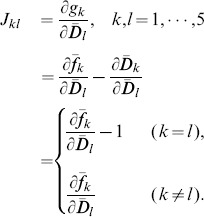
(14)


Because it is difficult to calculate Eq. (14) directly, we estimated the Jacobian matrix at 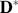 (denoted as 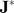 hereafter) using the following approximate form:
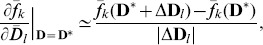
(15)where 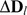 is a vector with an 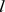-th element valued 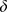 and the others zero, e.g., 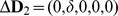. For the numerical calculation, 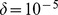 was used. We then calculated the eigenvalues for 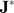, 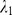, 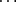, 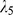. When 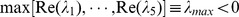, the equilibrium 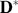 is determined as stable.

The above method is not appropriate to examine whether the V-shaped network (Fig. 8**H**) is globally stable because changing the V-shaped network (Fig. 8**G**) to other network types, such as complete or partial mesh (Fig. 8**A, D, E**, or **F**), requires at least two additional links. In Eq. (14), only a single additional link can be considered. Therefore, instead of calculating eigenvalues, we estimate a time constant 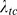 converging to 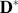 from a vicinity. We tested four combinations of deviations from the V-shaped equilibrium, 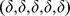, 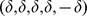, 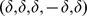, 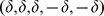. Finally, we defined the maximum time constant as 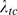.

**Figure 10:**
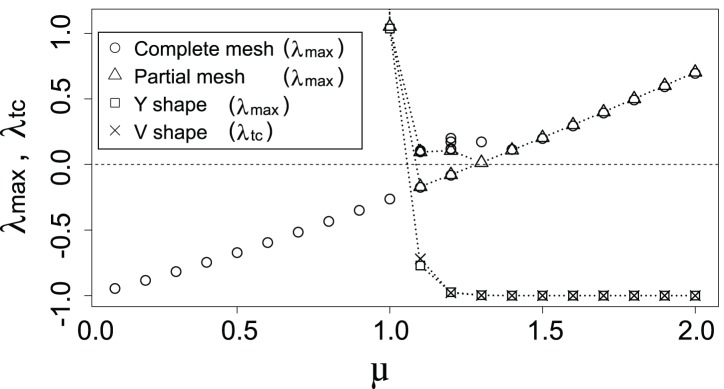
Maximum eigenvalues depending on 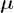 when 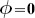. Circles, triangles, and squares, respectively, denote 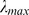 at the equilibria of complete mesh (Fig. 8**A**), partial mesh (Fig. 8**E**), and Y-shaped (Fig. 8**G**). Crosses represent 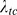 for V-shaped (Fig. 8**H**) networks.

Figure 10 summarizes the dependence of the maximum eigenvalues 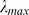 (or 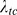 for the V-shaped network) on the parameter 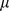. The single stable equilibrium, complete mesh, is found in the region of 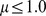. The complete mesh remains stable over 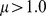 followed by participation of the Y-shaped, V-shaped, and partial mesh networks. The complete and partial meshes become unstable when 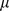 exceeds 1.3. The stability change from complete mesh, via partial mesh, to Y-shaped or V-shaped network resembles that of the larger network (Fig. 3). However, no significant difference can be found in the features of the stability among different phase-lags 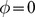, 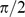 and 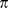 (Fig. S3) while appearance of Y-shaped or V-shaped network apparently depends on 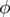 in the larger network, as seen in Fig. 4. The dependence would be caused by the difference in the basin sizes between the Y-shaped and V-shaped networks. Figure 9 shows the observation ratio of the Y-shaped and the V-shaped networks. Both types are always observed but the ratio of the Y-shaped network increases in accordance with 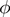. The change in basin size depending on 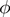 could explain the observation that the Y-shaped network is more frequently observed in anti-phase lag in the larger network, as seen in Fig. 4.

### Summary and Conclusion

In this paper, we proposed using the *Physarum* algorithm to design transportation network topologies and traffic distribution under oscillating conditions. The results of numerical experiments indicate that this approach is valid and has the following benefits:

1. Only one parameter 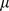 can control the morphology of the network. The client using the network can choose a particular parameter according to which they consider to be the most important among loss, cost, and vulnerability.
2. By introducing oscillating condition, building and/or maintenance cost is reduced to a maximum of 80% that of cases in which conditions are static.
3. Phase lag among outputs results in a wide variety of network morphology when 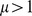 (sigmoidal growth in the conductance).

Table 2 summarizes the first item. Partial mesh can be recommended when the client requests a system with loss, cost, and vulnerability well-balanced. The third index, vulnerability, should be noted when considering power grids. The meshed network has a low vulnerability index but it includes loop connections, which are prone to cascading failure problems. When some nodes or links in a meshed network are damaged, the current that would normally go through those links must be distributed to the surrounding links. However, if the current goes beyond the capacity of the surrounding links, the damage propagates rapidly to the outer surrounding links. This results in large-scale blackouts [10], [11]. Considering these phenomena, V-shaped and Y-shaped networks are recommended rather than partial mesh. For railroads and highways, in which cascades need not be considered, partial mesh can be recommended. The cascading problem was not treated as a performance function in this paper because, for the sake of simplicity, the capacity of the current for each link was not considered. This will be dealt with in future work.

**Table 2:** Evaluation of the network type for each item.

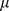	Network\Evaluation	Loss	Cost	Vulnerability	Cascading
<1.0	Complete mesh	A^+^	C	A^+^	C
1.0–1.4	Partial mesh	B	A	A^+^	C
>1.4	V-shaped orY-shaped	B or C	A^+^	C	A^+^

A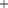: best, A: good, B: acceptable, C: bad.

For the second item, if a client considers the reduction of power loss more important than building and maintenance cost, a network that is designed under static conditions is recommended. The recommendation can be reversed by considering the third item, phase lag. Then, the problem of loss can be overcome.

**Figure 11:**
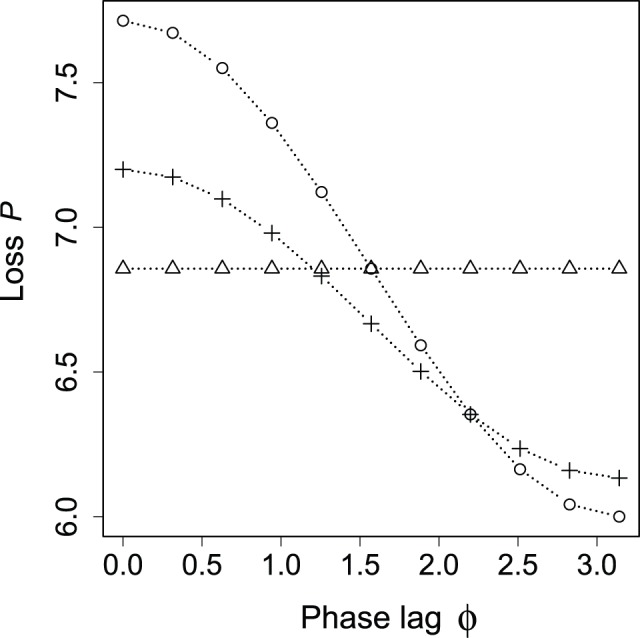
Relation between loss 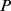 and 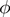 of small network. Circles, triangles, and crosses respectively denote Y-shaped network (Fig. 8**G**), V-shaped network (Fig. 8**H**), and complete mesh (Fig. 8**A**). The total volume ( = cost) for each network is normalized by that for the complete mesh so that the three networks are made with the same cost.

For the third item, the Y-shaped network is observable more frequently than the V-shaped network as the phase lag gets larger when 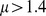. This topological selection delivers a maximum of 20% loss reduction to the system. Notably, the loss decreases when the lag approaches anti-phase away from in-phase, as shown in Fig. 11. This result theoretically supports a justification of the “peak shift” action developed in Japan for reducing electric power after the Fukushima nuclear disaster in 2011. The peak shift action shifts usage of electricity from on-peak to off-peak periods. This allows the electric power consumption in power grid systems to flatten during the day and to be reduced in peak periods. By introducing this action, the number of standby power plants can be reduced: Such the plants, e.g., thermal ones, are on standby to regulate power generation flexibly and to avert power shortages in peak periods. Our results suggest that peak shift action contributes to a reduction in not only the number of standby power plants but also in power loss in the grid.

Natural systems may gain advantages by self-organizing their network. Argentine ants are known to make supercolonies, which consist of multiple colonies with a single family. They form V-shaped or Y-shaped trails connecting the multiple colonies [12]. Army ants build dendritic trails–large-scaled Y-shaped branching structures [13]. Tao et al. showed, by a computer simulation, that virtual ants building Y-shaped trails can gain more food than those building V-shaped trails when the foods appear in anti-phase at two sites [14]. By considering the number of ants as cost, the result can be interpreted as the ants selectively building Y-shaped networks under constraints of constant cost and fluctuating environment. As a result of the selection, the ants can convey food with minimum loss. Of course, it is known that slime mold, the model organism inspires the *Physarum* algorithm itself, constructs Y-shaped, V-shaped and dendritic networks depending on environmental conditions [15]–[17]. The slime mold and the ants selected the optimum way without any systematic plan long before humans analyze such as these.

## Supporting Information

Figure S1 **Full list of network topologies with oscillating condition.** The topology number corresponds to that of Figs. 2 and 3.
(EPS)

Figure S2
**Relation between the types of partial mesh and** 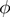 **.** 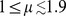. The type number corresponds to that of Fig. S1.(EPS)

Figure S3
**Maximum eigenvalues depending on** 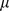 **when** 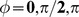 **.** 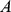 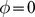. 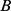 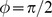. 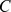 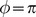. Circles, triangles, and squares, respectively, denote 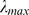 at the equilibria of complete mesh (Fig. 8A), partial mesh (Fig. 8E), and Y-shaped (Fig. 8G). Crosses represent 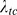 for V-shaped (Fig. 8H) networks.(EPS)

File S1 **Footnotes.**
(PDF)
