# Supplementary material for: Transportation Network with Fluctuating Input/Output Designed by the Bio-Inspired Physarum Algorithm
Source: PLoS One. 2014 Feb 26;9(2):e89231. doi: 10.1371/journal.pone.0089231 (PMC3935870; doi:10.1371/journal.pone.0089231)
Supplement: File S1 — Footnotes. (PDF) [file pone.0089231.s004.pdf]

Supporting Information: File S1

S. Watanabe and A. Takamatsu

1 Effect of output frequency

The numerical calculations of this system were performed over the frequency range $2\pi \times 10^{-2} \leq \omega \leq 2\pi \times 10^{7}$. When $2\pi \times 10^{-1} \leq \omega \leq 2\pi \times 10^{7}$, the conclusions in the main text are valid, except for the oscillatory behavior of the converged $D_{ij}(t)$ detailed below. Interestingly, the network topology still depends on phase-lag ϕ in two oscillating outputs, even when the frequency is very high ($\omega = 2\pi \times 10^{7}$) against time constant of D-degeneration (estimated as 1 from Eq. (4)). This could be caused by nonlinearity of the growth function, Eq. (5), where the dependence of the time-average of the flux Q on ϕ survives in this system at least in this frequency range (see also Section 4 in File S1). Conversely, when the frequency is extremely low, such as $\omega = 2\pi \times 10^{-2}$, the system can follow the slow change of flux. Then, the converged network topology differs from that for higher frequency, as shown in Fig. A in File S1. The system exhibits a V-shaped network when $\phi = 0$ (Fig. A(a)) and a Y-shaped network when $\phi = \pi$ (Fig. A(b)) in the higher frequency, which is consistent with the results in the main text. However, the system always exhibits a V-shaped network in the lower frequency $\omega = 2\pi \times 10^{-1}$ (Fig. A(c)–(d)).

To be more specific about the oscillatory behavior of converged $D_{ij}(t)$, the mean value (equivalent to $\tilde{D}_{ij}$) does not depend on frequency but the amplitude does, as illustrated in Fig. A and summarized in Fig. B.

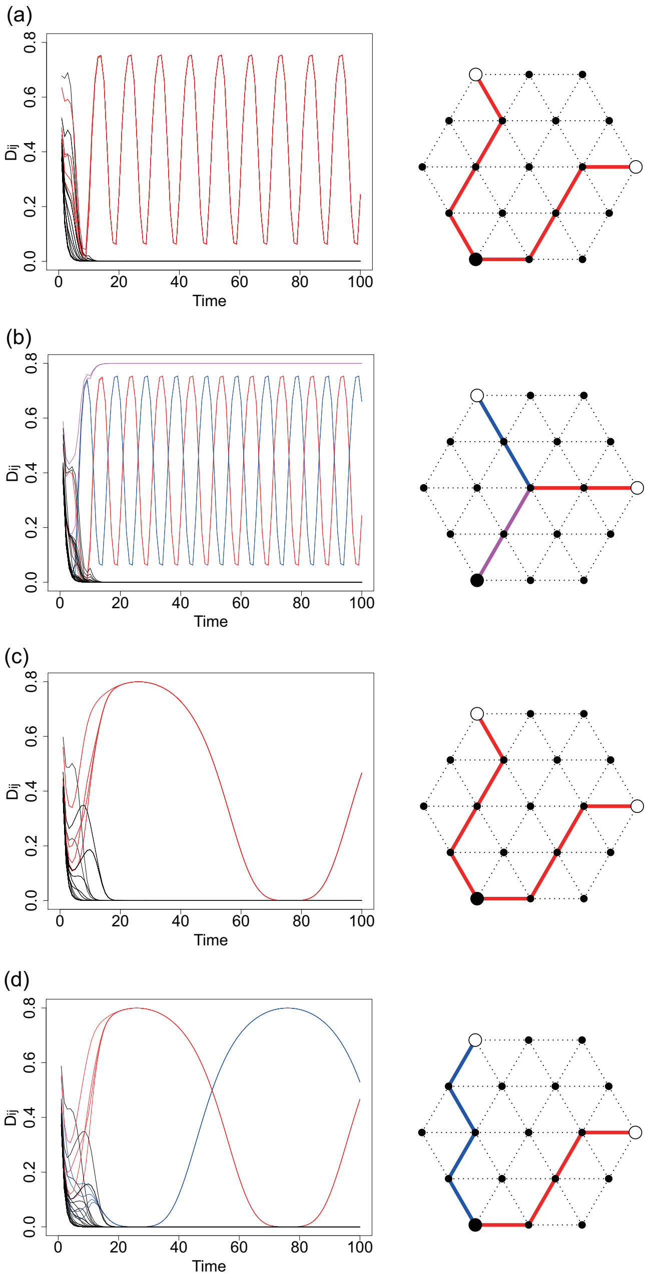

Figure A: Effect of output frequency. (a) $\omega = 2\pi \times 10^{-1}, \phi = 0$. (b) $\omega = 2\pi \times 10^{-1}, \phi = \pi$. (c) $\omega = 2\pi \times 10^{-2}, \phi = 0$. (d) $\omega = 2\pi \times 10^{-2}, \phi = \pi$. Left panels show time-variable D_{ij}. Right panels represent the converged network topologies. The colors of the plots correspond to those in the network diagrams.

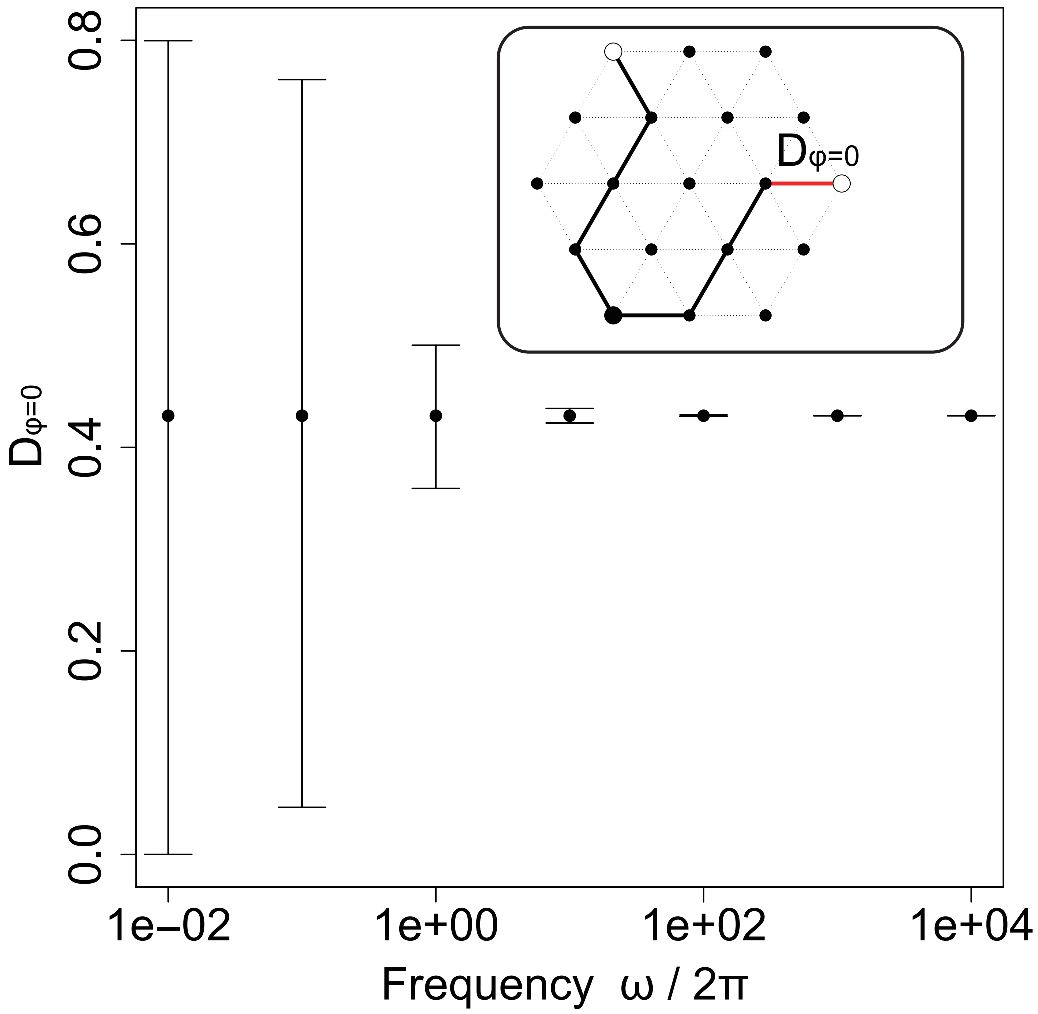

Figure B: Relation between converged $D_{ij}(t)$ and frequency. Closed circles denote time average of the converged $D_{ij}(t)$ over a period (equivalent to $\tilde{D}_{ij}$). Bar represents the amplitude of oscillation in $D_{ij}(t)$. The data are obtained for the links colored red in the inset diagram. The phase lag was set at $\phi = 0$.

2 The converged value $\tilde{D}_{ij}$ versus time-variable $D_{ij}(t)$ on estimation of performance functions.

In the numerical calculations, converged value $\tilde{D}_{ij}$ is used instead of time-variable $D_{ij}(t)$ for application to man-made systems such as practical power grids because wires with time-variable conductance are not realistic. However, for application to natural systems such as slime mold and ant trails, the time-variable conductance is possible. We tested the case of $D_{ij}(t)$ instead of $\tilde{D}_{ij}$ in the calculation of Eqs. (9) and (10) using the following definition,

$$P_t = \frac{1}{T}\int_0^T \left\{ \sum_{ij} Q_{ij}^2(t) \frac{L_{ij}}{D_{ij}(t)} \right\} dt \ , \tag{S1.1}$$

$$B_t = \sum_{ij} L_{ij} D_{ij}(t) \ , \tag{S1.2}$$

and obtained virtually the same result. The relative residuals between the two calculation methods were a maximum of $|P_t - P|/P = 5.6 \times 10^{-5}$ for loss P, and a maximum of $|B_t - B|/B = 2.1 \times 10^{-3}$ for cost B. These are sufficiently small that the results in the main text are still valid for the case of the time-variable conductance.

3 Comparison of performance functions starting from homogeneous and non-homogeneous initial conditions.

Performance functions are calculated for the systems starting from the homogeneous and non-homogeneous initial conditions of D_{ij}. The results in the case of the non-homogeneous condition hold almost the same feature as in the case of the homogeneous condition, as evidenced in Figs. C-E. A small difference is observed only in P. The non-homogeneity causes multiple values of P at a certain combination of μ and ϕ, for example, multiple crosses at $\mu = 3.5, 4.0, 4.5, 5.0$ in Fig. C(a). The multiplicity is derived from the difference in the network topology.

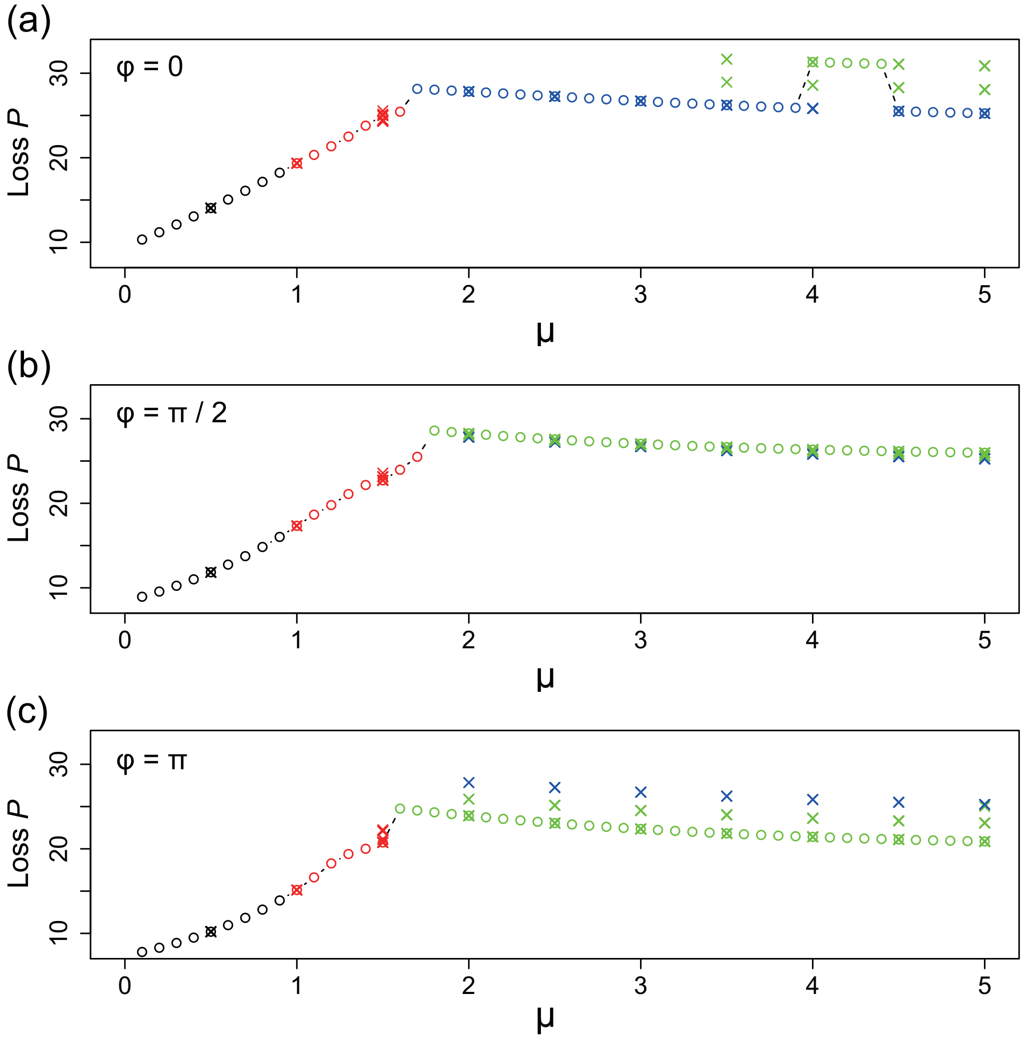

Figure C: Loss. The phase lags are set as (a) $\phi = 0$, (b) $\phi = \pi/2$, and (c) $\phi = \pi$. Color of plots represents network topology: Black, red, blue, and green denote, respectively, mesh, partial mesh, V-shaped, and Y-shaped networks. Circles are results starting from the homogeneous initial conditions of D_{ij}. Crosses are results starting from the non-homogeneous initial conditions, where D_{ij} are distributed according to a normal distribution with mean 1.0 and standard deviation 0.1. One hundred samples for each parameter set of μ and ϕ were tested for the non-homogeneous conditions.

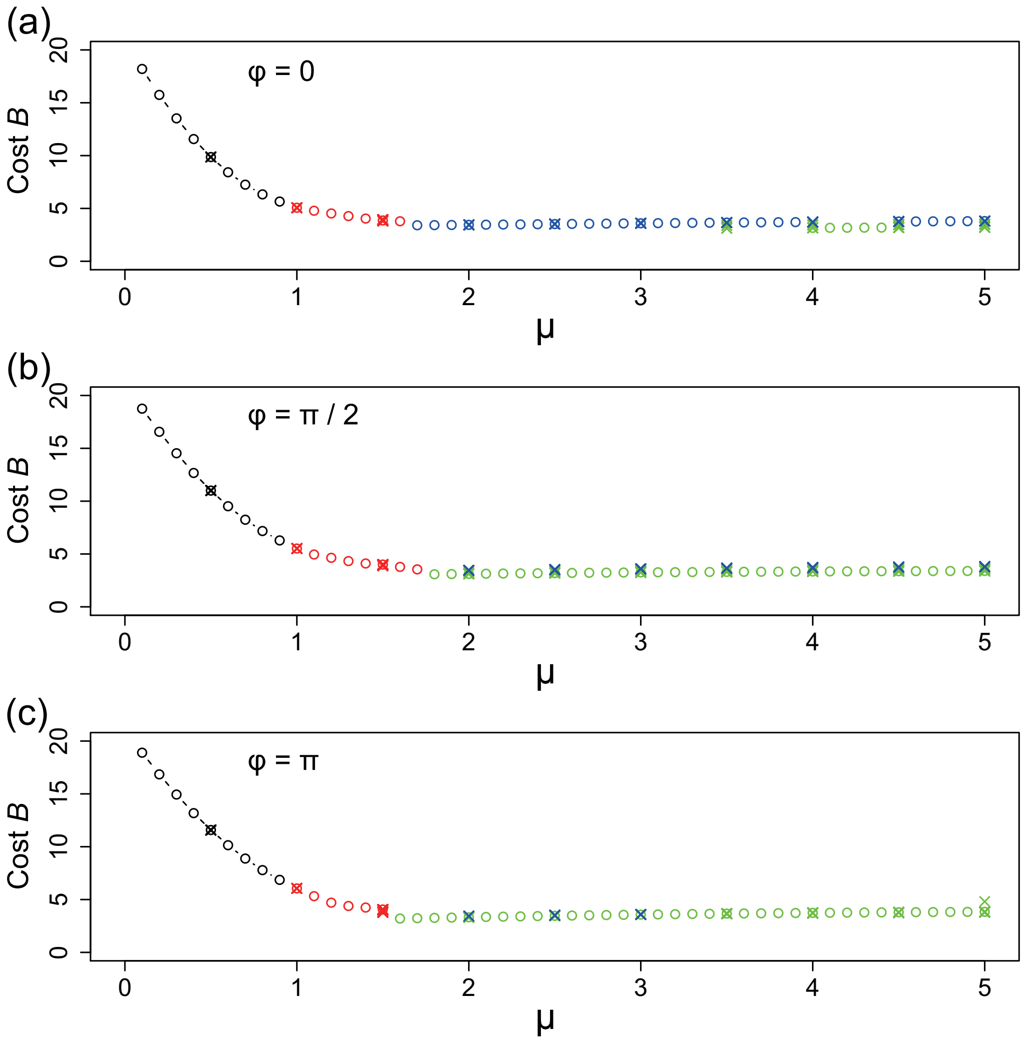

Figure D: Cost. Notations and conditions of numerical calculation are the same as those of Fig. C.

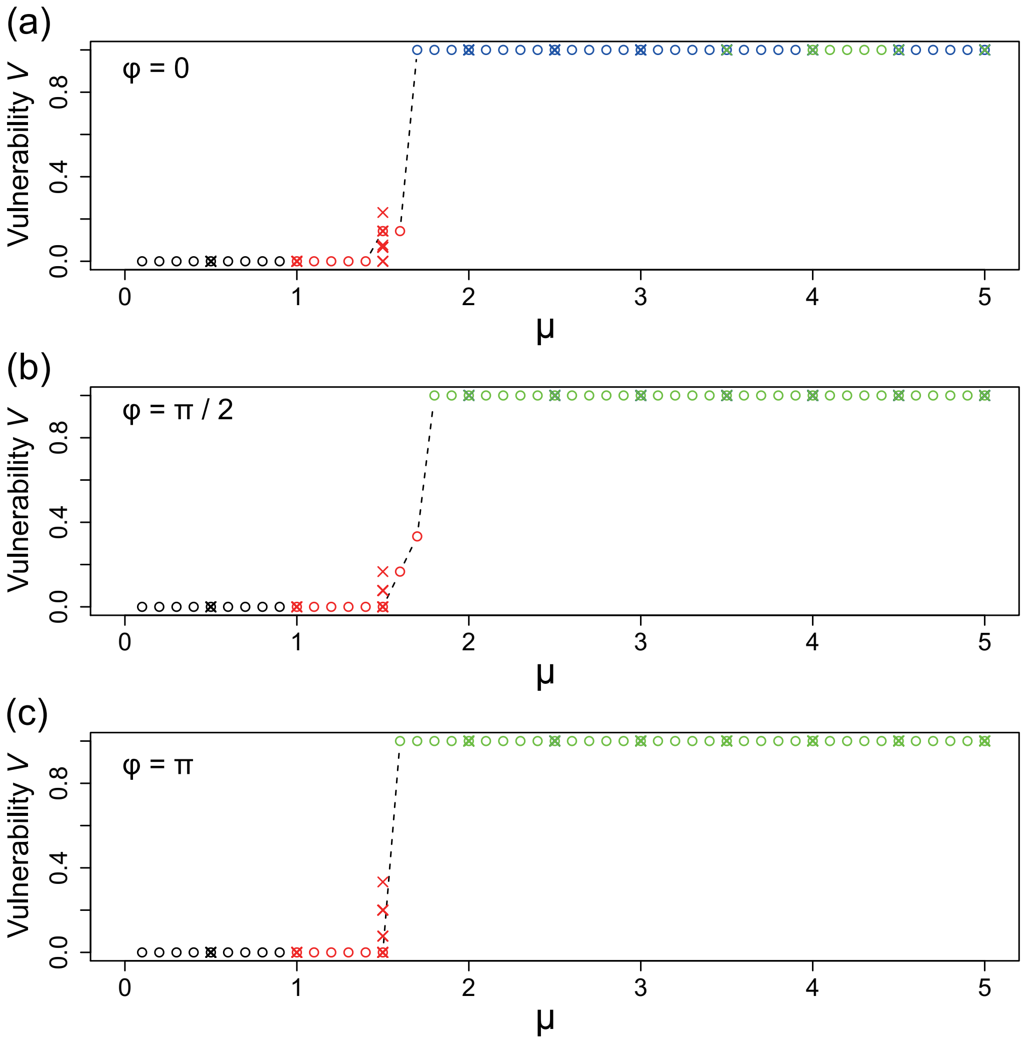

Figure E: Vulnerability. Notations and conditions of numerical calculation are the same as those of Fig. C.

4 Dependence of integration of $f(|Q|)$ on ϕ

In the calculation of Eq. (13) in the main text, the time-averaged value of $f(|Q|)$ over a period depends on ϕ because the function is nonlinear. The flux Q_i at each link l_i is the sum of those originating from out_1 and out_2, which are respectively denoted as $q_i^{out_1} = a_i(1+\sin\omega t)$ and $q_i^{out_2} = b_i\{1+\sin(\omega t+\phi)\}$. Then $|Q_i| = a_i + b_i + a_i \sin(\omega t) + b_i \sin(\omega t + \phi)$. The parameters a_i and b_i are certain constants calculated for each parameter condition of μ and ϕ.

First, assume the linear case: $f(|Q|) = |Q|$, the integration over a period is as follows:

$$\begin{aligned}\int_0^T f\{|Q_i(t)|\}dt &= \int_0^{2\pi} \{a_i + b_i + a_i \sin(\theta) + b_i \sin(\theta+\phi)\}d\theta \\ &= 2\pi(a_i + b_i)\ ,\end{aligned} \tag{S1.3}$$

which is not dependent on ϕ.

Second, assume the simplest example for the nonlinear case: $f(|Q|) = |Q|^2$, the integration over a period is as follows:

$$\begin{aligned}\int_0^T f\{|Q_i(t)|\}dt &= \int_0^{2\pi} \{a_i + b_i + a_i \sin(\theta) + b_i \sin(\theta+\phi)\}^2 d\theta \\ &= \pi\{3a_i^2 + 3b_i^2 + 4a_ib_i + 2a_ib_i\cos\phi\}\ .\end{aligned} \tag{S1.4}$$

Now the integration depends on ϕ.

Last, consider the case of Eq. (13),

$$\begin{aligned}\int_0^T f\{|Q_i(t)|\}dt &= \int_0^{2\pi} \frac{|Q_i|^\mu}{1+|Q_i|^\mu} d\theta \\ &= \int_0^{2\pi} f(\theta,\phi)d\theta\ ,\end{aligned} \tag{S1.5}$$

where

$$f(\theta,\phi) \equiv \frac{[a_i\{1+\sin\theta\} + b_i\{1+\sin(\theta+\phi)\}]^\mu}{1+[a_i\{1+\sin\theta\} + b_i\{1+\sin(\theta+\phi)\}]^\mu}. \tag{S1.6}$$

Although it is difficult to integrate Eq.(S1.6) analytically, the dependence of the function $f(\theta,\phi)$ on ϕ can be captured intuitively from $\theta - f(\theta)$ plots with different phase lags ϕ, as shown in Fig. F. Thus, it is obvious that the integration of Eq.(S1.6) over a period depends on ϕ. The values $\int_0^{2\pi} f(\theta,\phi)d\theta$ were numerically estimated as $3.844, 4.379, 5.027$, respectively, when $\phi = 0, \pi/2, \pi$, and $\mu = 2.0$, $a_i = 1$, $b_i = 1$.

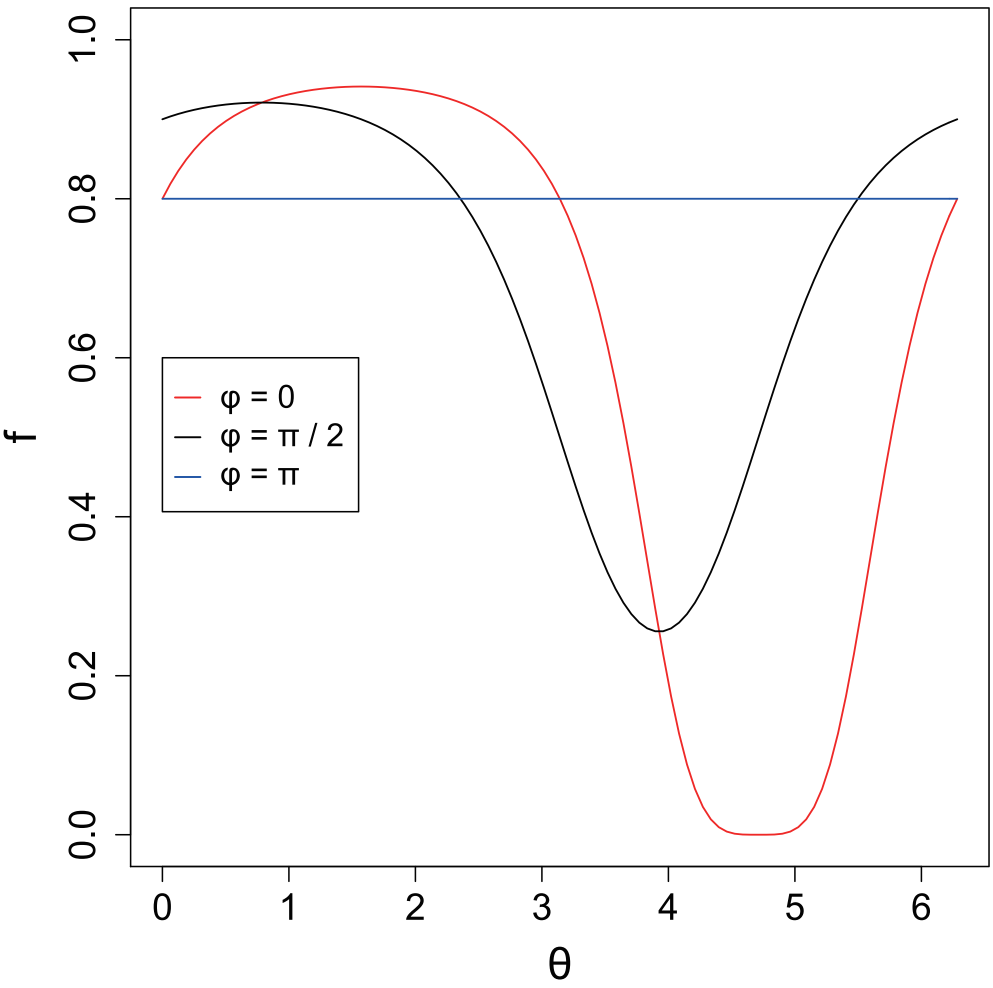

Figure F: Growth function $f(\theta, \phi)$ with different phase lags ϕ. $\mu = 2.0$, $a_i = 1$, $b_i = 1$.
